# Enhancing the uptake of intermittent preventive treatment for malaria in pregnancy: a scoping review of interventions and gender-informed approaches

**DOI:** 10.1186/s12936-025-05275-z

**Published:** 2025-02-18

**Authors:** Irene A. Kretchy, Deborah Atobrah, David A. Adumbire, Samuel Ankamah, Theodosia Adanu, Delali M. Badasu, Benjamin K. Kwansa

**Affiliations:** 1 Department of Pharmacy Practice and Clinical Pharmacy, University of Ghana School of Pharmacy, Accra, Ghana; 2 Centre for Gender Studies and Advocacy, University of Ghana, Accra, Ghana; 3 Institute of African Studies, University of Ghana, Accra, Ghana; 4 Regional Institute for Population Studies, University of Ghana, Accra, Ghana; 5 University of Ghana Library System, University of Ghana, Accra, Ghana

**Keywords:** Malaria in pregnancy, IPTp, Interventions, Sulfadoxine-pyrimethamine, Gender roles, Sub-Saharan Africa, Malaria-endemic regions, Malaria prevention and control

## Abstract

**Background:**

Malaria infection in pregnancy is a critical determinant of maternal and neonatal health outcomes in endemic regions. Intermittent preventive treatment of malaria in pregnancy (IPTp) using sulfadoxine-pyrimethamine has been recommended by the World Health Organization (WHO), but its uptake remains low because of factors such as gender norms and expectations. However, interventions to optimize IPTp uptake, especially in malaria-endemic regions, have resulted in a decline in malaria during pregnancy, maternal and neonatal mortality, low birth weight, and placental parasitaemia. This scoping review aimed to synthesize evidence on IPTp uptake, particularly emphasizing gender-related strategies.

**Methods:**

The modified version of Arksey and O'Malley's framework and the Preferred Reporting Items for Systematic Reviews and Meta-Analysis Extension for Scoping Reviews (PRISMA-ScR) were adopted for this review. Documents were retrieved from the following electronic databases and search engines: scopus, Web of Science, CINAHL Complete (EBSCO), PubMed, WHO, Global Index Medicus, and Google Scholar. The titles and abstracts of the publications were independently screened via Rayyan review management software, and the data were organized using the reach, effectiveness, adoption, implementation, and maintenance (RE-AIM) framework and gender analysis matrix.

**Results:**

A total of 32 studies met the inclusion criteria. The most reported criterion was the effectiveness of the interventions, which demonstrated an increase in IPTp uptake after the intervention. The gender analysis framework revealed that involving both men and women in decision-making processes, empowering women, and promoting shared roles could improve the success of IPTp interventions.

**Conclusions:**

Interventions to increase IPTp uptake should be targeted at empowering women through education, increasing financial independence, and making decisions about their health.

## Background

Despite increased efforts by both private and public actors to prevent and control malaria, infection remains a major public health concern across the globe, especially in malaria-endemic regions. Malaria accounts for more than 245 million deaths, the majority of which occur in low- and middle-income countries (LMICs), with pregnant women being the most affected demographic group.

Malaria in pregnancy (MiP) is a major public health and policy concern, particularly in sub-Saharan Africa (SSA), due to its cascading effect on maternal and child health outcomes. Within the malaria-hyperendemic areas of the SSA region, pregnant women, young children, and infants are the subpopulation groups most vulnerable to malaria infection [1]. Approximately 125 million women in malaria-endemic countries become pregnant annually. In the SSA region alone, 25 million pregnant women are at risk of malaria infection annually and require prophylaxis [2].

Malaria in pregnancy (MiP) contributes significantly to maternal anaemia, death, and severe risks for unborn children, including fetal loss, low birth weight and premature delivery, in many SSA countries [3]. In 2022, there were 249 million malaria cases worldwide, resulting in 608,000 deaths. Among these, 76% were children under the age of five, equating to over 1000 child deaths per day [4]. In 2023, there were 263 million malaria cases and 597, 000 malaria deaths worldwide. In the WHO African region, 246 million malaria cases were reported with 569 000 deaths [5]. Infant mortalities in the malaria-endemic zones of Africa are linked to MiP which contributes to 12–20% of stillbirths in sub-Saharan Africa [6]. Factors contributing to MiP in SSA include maternal immunity, parasite density, parity, inadequate antenatal care services, drug misuse and abuse, intermitted preventive treatment drug failure and resistance [7, 8].

To prevent malaria in pregnancy, the World Health Organization (WHO) recommends a multipronged approach that includes both preventive and curative measures [9]. The recommended interventions for preventing and controlling MiP include the promotion and use of insecticide-treated nets (ITNs) and appropriate case management with prompt and effective treatment [9]. In malaria-endemic areas in Africa, IPTp with sulfadoxine-pyrimethamine (IPTp-SP) was recommended by the WHO in 2012 for all pregnant women in their second trimester. This prophylaxis is administered during antenatal visits to reduce the disease's impact on mothers, fetuses, and newborns [2]. IPTp-SP should ideally be dispensed as a directly observed therapy in a single dose of three tablets, each containing 500 mg of sulfadoxine and 25 mg of pyrimethamine, for a total of 1500 mg of sulfadoxine and 75 mg of pyrimethamine. IPTp intervention is initiated in the second trimester and administered at intervals of at least one month, with a recommended minimum of three doses throughout the pregnancy.

An analysis of intervention trials with IPTp revealed an approximately 38% reduction in the risk of severe maternal anaemia, a 43% reduction in low birth weight, and a 27% reduction in perinatal mortality among paucigravidae [9, 10]. Other previous trials suggest that successful malaria infection prevention with chloroquine prophylaxis or IPTp reduces the risk of low birth weight by 43% [11].

Despite the importance of IPTp in malaria prevention and several years after its implementation, MiP remains a challenge in most malaria-endemic countries because of the low uptake of IPTp. A study by Pons-Duran et al. (2021) conducted in four sub-Saharan African countries—the Democratic Republic of Congo (DRC), Madagascar, Mozambique, and Nigeria—revealed that over two-thirds of pregnant women who attended at least four antenatal care visits during pregnancy received fewer than the three doses of IPTp recommended by the WHO. The low uptake of IPTp in SSA is associated with individual, community, and country-level factors, including personal and cultural beliefs, systemic factors, gender norms, the household wealth index, spouses’ educational level, and media exposure [12, 13]. Interventions to optimize IPTp uptake have, however, resulted in a decline in MiP episodes, maternal and neonatal mortality, low birth weight, and placental parasitaemia [14–16]. This review, therefore, aimed to synthesize existing evidence on malaria IPTp interventions for pregnant women and assess the reach, effectiveness, adoption, implementation, maintenance, and gender-related support strategies to scale up the prevention of MiP in alignment with ongoing malaria elimination efforts.

## Methods

### Search strategy and selection criteria

**Fig. 1:**
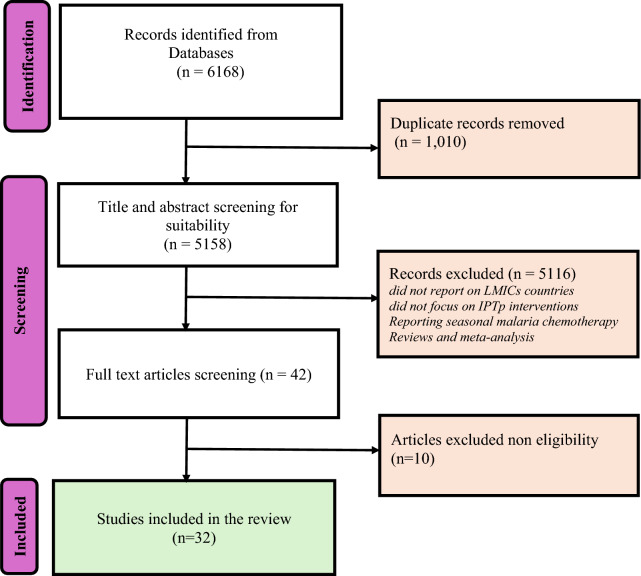
Flow diagram of scree*n*ing and selection of studies

This review was guided by the methodological framework for scoping reviews developed by [17] and modified by [18], with the primary goal of mapping the literature on the types, nature, and impact of interventions to improve malaria IPTp, with a focus on gender-related factors and areas for further research. The review steps involved identifying the research questions; identifying relevant studies; selecting relevant studies; charting and synthesizing data; and collating, summarizing, and reporting relevant studies. The preferred reporting item for systematic reviews and meta-analyses extension for scoping reviews (PRISMA-ScR) was used [19], as shown in Fig. 1. The review protocol is registered in the open science framework (ref ID: https://doi.org/10.17605/OSF.IO/V5D7U).

Three research questions were formulated to guide the literature search: (1) What are the interventions and populations of focus (individual, household, facility, and community) utilized to increase access to and uptake of IPTp during pregnancy; (2) how does the intervention align with the RE-AIM framework; and (3) how is gender incorporated in these interventions through the use of gender indicators, such as acknowledgement of gender disparities, addressing gender barriers, utilization of sex-disaggregated data, gender analyses, and women's empowerment?

The following electronic databases and search engines were searched for relevant documents between March 2024 and June 2024: Scopus, Web of Science, CINAHL Complete (EBSCO), PubMed, WHO, Global Index Medicus, and Google Scholar. Grey literature was sourced from Google Scholar, while hand searches were also conducted to source information. The key concepts used in the search included malaria, IPTp, pregnant women, low- and middle-income countries, their synonyms, and related terms developed via Medical Subject Headings (MESH). The concepts were identified via the population, concept, and context (PCC) approach for pregnant women, malaria IPTp, and low- and middle-income countries. The search terms were combined to create search queries via Boolean operators and revised as required for maximum results. The detailed search strategy is provided in the Supplementary Information (Supp. File 1). Studies published between 2004 and 2023 were included because IPTp was first introduced in 2004 [20].

The studies were identified and screened using the following eligibility criteria: studies published in English; documents focusing on IPTp interventions or strategies that were used to prevent MiP; comparative or non-comparative intervention studies adopting quantitative, qualitative, or mixed methods approaches; and publications from LMICs. No study was excluded based on methodological quality or bias risk, in line with other scoping reviews [21]. Documents reporting seasonal malaria chemotherapy, other malaria interventions such as ITN use, non-MiP prevention interventions, and reviews and meta-analyses were excluded. While there are ongoing malaria intervention programmes, including Roll Back Malaria, the Global Malaria Programme, the United to Beat Malaria, and the United Nations Foundation, as well as the K4Health portal, Health Compass, and Community Case Management (CCM), this review included only journal-published articles on IPTp interventions for pregnant women.

### Data extraction and synthesis

Three independent reviewers (IAK, BKK, and DA) completed the title and abstract screening, full-text review, and data extraction with consensus meetings with the research team to address any disagreements. The search results were imported into EndNote version 21. Following the removal of duplicate documents, study titles and abstracts were screened for eligibility via Rayyan web application software. The full texts of all potentially relevant studies were obtained and screened to confirm their eligibility.

### Quality assessment

Information from the selected documents was recorded via a data extraction template (Table 1), which included data on the authors, year of publication, country of study, type of intervention, intervention population characteristics, intervention duration, and IPTp outcomes (See Table 2). The RE-AIM framework was used to evaluate the reach, effectiveness, adoption, implementation, and maintenance of interventions [22]. The five-stage reach, effectiveness, adoption, implementation, and maintenance (RE-AIM) framework [22, 23], was adopted and was based on an iterative approach of refining the study selection for data charting. The RE-AIM framework was developed over two decades ago to address the longstanding challenges and delays in translating scientific evidence into practical applications and policies [24]. The framework is often used in the planning and evaluation of health interventions to ensure the generalization and facilitation of the translation of research into practice [25]. It has become one of the most widely used frameworks for planning and evaluation in public health, behavioural science, and implementation science [25]. The framework has also been applied across various settings, populations, and health domains, including clinical, community, and corporate contexts [22–24, 26].

The review used the RE-AIM framework as an analytical framework because it provides a structured and multidimensional approach to evaluate public health interventions. Specifically, it allows for an assessment of the effectiveness of interventions to increase IPTp uptake and their reach, adoption, implementation, and maintenance. However, the study did not specifically set out to look for articles or projects that used the RE-AIM framework. The framework aligns with the objectives of this review, which focuses on understanding both the implementation processes and the outcomes reported in the included studies. Additionally, the review analyses focused on the available evidence on interventions to increase IPTp uptake, as reported in the included articles. While some studies have provided a comprehensive description of the intervention, others have focused on specific aspects such as social dimensions of community delivery of IPTp [27].

The following variables for data extraction were selected to align with each dimension of the RE-AIM framework: (1) reach: inclusion criteria and primary outcome; (2) effectiveness: type and intervention outcome; (3) adoption: intervention development, country of intervention, and intervention setting; (4) implementation: intervention dosage, roles of various intervention implementers, barriers to intervention implementation, and intervention design; and (5) maintenance: availability of schedule of postintervention data collection.

Gender-related considerations of the interventions were extracted via an adaptation of the gender analysis matrix [28]. This gender analysis matrix was used to conduct a systematic gender analysis of the various interventions selected. The matrix included questions in each cell, including access to funds needed for IPTp treatment; roles and practices affecting IPTp uptake; social norms, values, and beliefs in the community; IPTp uptake decision-making power; and autonomy of IPTp uptake. The questions were posed for further reflection and analysis related to the intersection of each topic-specific domain and the gender analysis domain. Using this gender framework, these questions address the different ways in which gender power relations manifest as inequities and how they may affect the perception and utilization of IPTp [28].

The gender matrix was chosen for this study review because of its robust framework to systematically integrate gender considerations into the analysis of infectious disease interventions. This review adopted the matrix for this paper because while initially conceived for infectious disease outbreaks, its application here allows us to critically assess the gender dimensions of IPTp interventions, which are similarly affected by social, cultural, and systemic gender inequities.

This paper recognizes that the studies included in the review may not have explicitly used the gender analysis matrix proposed by [28] to assess the gender dimensions of their projects. However, the gender matrix adopted in the review was used only as an analytical framework. The paper thus acknowledges that not all the aspects of the gender analysis matrix may have been reported in the articles, even if they were accounted for in the project design or implementation. This confirms the potential variability in the depth of gender-related reporting across the included studies.

## Results

**Fig. 2:**
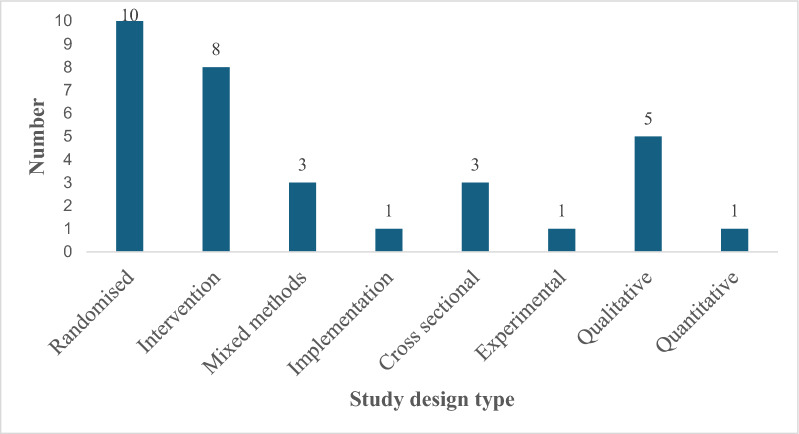
Number of studies included according to design

A total of 6168 studies were identified in the initial search, of which 1010 duplicates were removed. After screening the titles and abstracts, 42 full texts were assessed for eligibility (Fig. 2). Of these, 32 (0.51%) met the inclusion criteria and were included in the final analysis. The eligible studies were published between 2007 and 2024. A wide variety of study designs were used in the interventions. The highest proportion (32%) of the studies were randomized controlled trials. There was one implementation study and two quantitative and experimental studies. In total, they accounted for nine percent (9%) of all the studies included in the analysis.

**Fig. 3:**
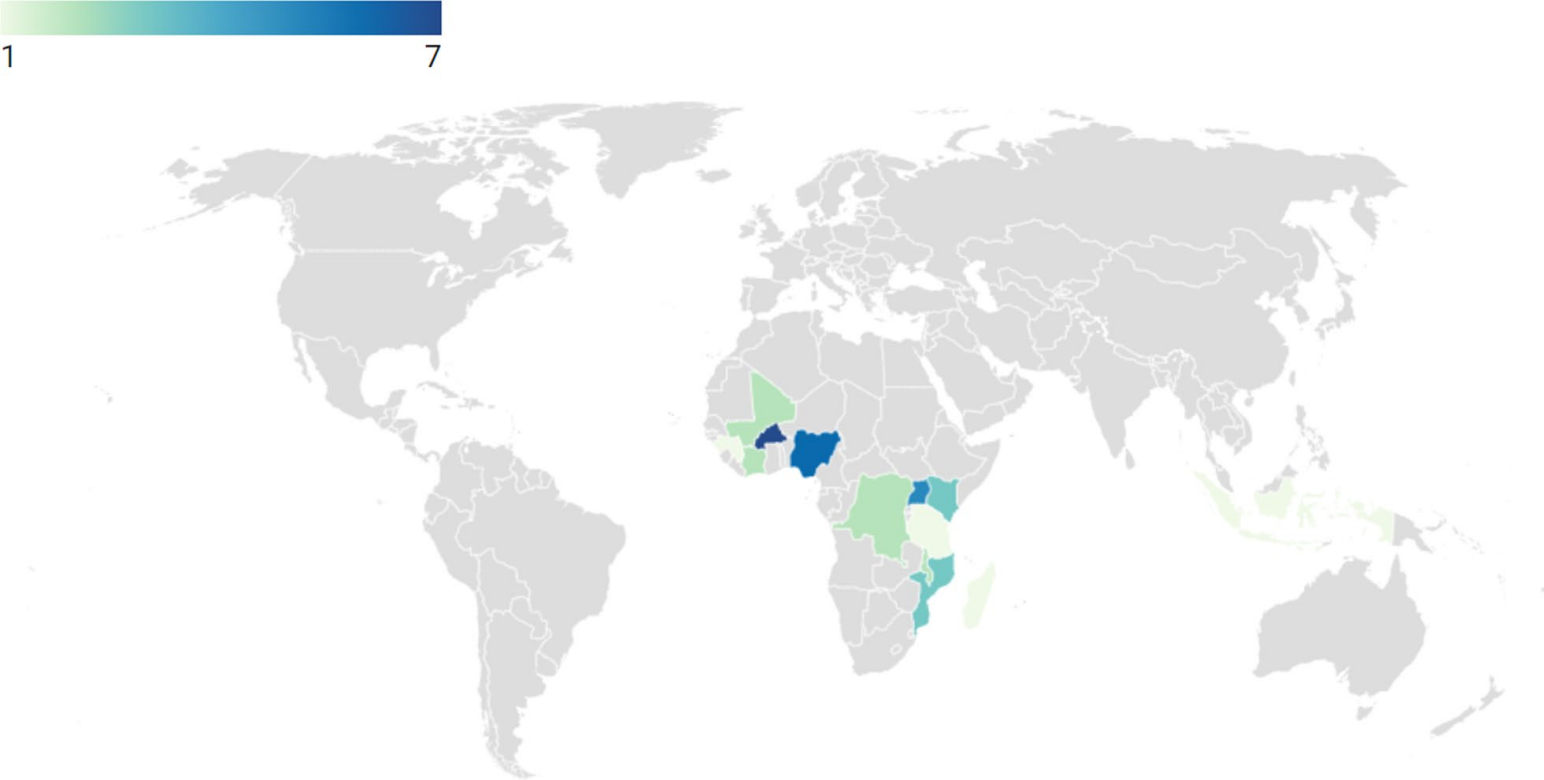
Countries where the included studies were carried out

The total population of the included studies was 169,818, consisting of different population subgroups, including pregnant women, adolescents, health workers, community leaders, stakeholders and health managers. Geographically, the studies encompassed diverse regions and countries in malaria-endemic areas, reflecting a generalized perspective on the topic. The included studies covered approximately thirteen (13) countries, with twelve of the countries in the African region (as shown in Fig. 3.)

The study settings, including clinical, educational, and community settings, varied; pregnant women (n = 4), health workers (n = 4), and the community (n = 18) were targeted. One intervention study focused on pregnant women and the community, whereas five focused on community and health workers. The duration of the interventions varied from one month, with the longest duration being at least 36 months (Table 1).

**Table 1:** Study details of interventions to increase IPTp

Article	Study design	Year published	Intervention location	Population of focus	Duration of intervention
Hildon et al., 2020 [13]	Multimethod	2020	Mozambique	Community members and key influencers	Not Reported
Hoyt et al., 2018 [14]	Qualitative study	2018	Eastern Indonesia	Health workers and Pregnant women	20 months
Msyamboza et al., 2009 [16]	Intervention study	2009	Malawi	Community	30 months
Enguita-Fernàndez et al., 2021 [27]	Qualitative Study	2020	Democratic Republic of Congo Madagascar, Mozambique and Nigeria	Community, Health Workers	4 months
Koita et al., 2024 [29]	Cluster randomized implementation trial	2024	Mali and Burkina Faso	Pregnant women	Not reported
Grietens et al., 2010 [30]	Randomized community-based trial	2010	Burkina Faso	Adolescents	36 months
Vanga-Bosson et al., 2011 [31]	cross-sectional survey	2011	Côte d'Ivoire	Pregnant women	6 Months
Mbonye et al. 2008 [32]	Intervention Study	2008	Uganda	Pregnant women	21 months
Odwe et al., 2023 [33]	Cross-sectional baseline survey	2023	Kenya	Women aged 15–49	2 months
Noguchi et al., 2020 [34]	Pragmatic, cluster randomized, controlled trial	2020	Nigeria and Kenya	Pregnant women	15 Months
Koné et al., 2023 [35]	Randomized controlled trial	2023	Abidjan, Ivory Coast	Health workers	12 months
Gies et al., 2009 [36]	Cluster-randomized trail	2009	Burkina Faso	Pregnant women	36 months
Flueckiger et al., 2019 [37]	Implementation study	2019	Guinea	Community	2 months
Rassi et al. 2018 [38]	Convergent mixed methods	2018	West Nile, Uganda	Health workers	8 months
Rubenstein et al., 2022 [39]	Cluster randomized, controlled trial	2022	Malawi	Pregnant women	21 months
Winskill et al., 2019 [40]	Quantitative analysis	2019	Sub-Saharan Africa	Community and health facility	Not reported
Ouédraogo et al., 2022 [41]	Randomized trial	2022	Burkina Faso	Pregnant women	13 months
Okeibunor et al., 2011 [42]	Interventional study	2011	Akwa Ibom, Nigeria	Community	26 months
Doumbia et al., 2021 [43]	Mixed method	2021	Mali	Pregnant women	3 months
Gutman et al. 2020 [44]	Randomized controlled trial	2020	Burkina Faso	Pregnant women	15 Months
Mbonye, Bygbjerg, & Magnussen, 2007 [45]	Intervention Study	2007	Uganda	Community	21 months
Gies et al., 2008) [46]	Intervention Study	2008	Burkina Faso	Community	24 months
Ouma et al., 2007 [47]	Cross-sectional survey	2007	Kenya	Pregnant women	5 months
Wolf et al., 2023 [48]	Intervention study	2024	Cameroon, Cote d’Ivoire, Ghana, Kenya,Mali, and Niger	Community	12 months
Mens, 2011 [49]	Intervention studies	2011	Edo State, Nigeria	Community/health workers	Not reported
Burke et al., 2021 [50]	Qualitative study	2021	Burkina Faso	Community Health workers and health facility workers	16 months
Orobaton et al., 2016 [51]	Intervention study	2016	Nigeria	Community, Health Workers	42 days
Graham & Ba-Break., 2013 [52]	Qualitative study	2013	Tanzania	Health Workers and Health Managers	1 month
Balami et al., 2019 [56]	Randomized controlled study	2019	North-eastern Nigeria	Pregnant women	6 months
Faye & Lugand, 2021 [57]	Qualitative study	2021	Democratic Republic of Congo, Nigeria and Mozambique	Healthcare providers, CHW and pregnant women,	5 months
Mbonye et al., 2013 [60]	Quasiexperimental study	2013	Uganda	Pregnant women	12 months
Hansen et al., 2012 [61]	Randomized controlled trial	2012	Southwestern Uganda	Community and Pregnant women	36 months

**Fig. 4:**
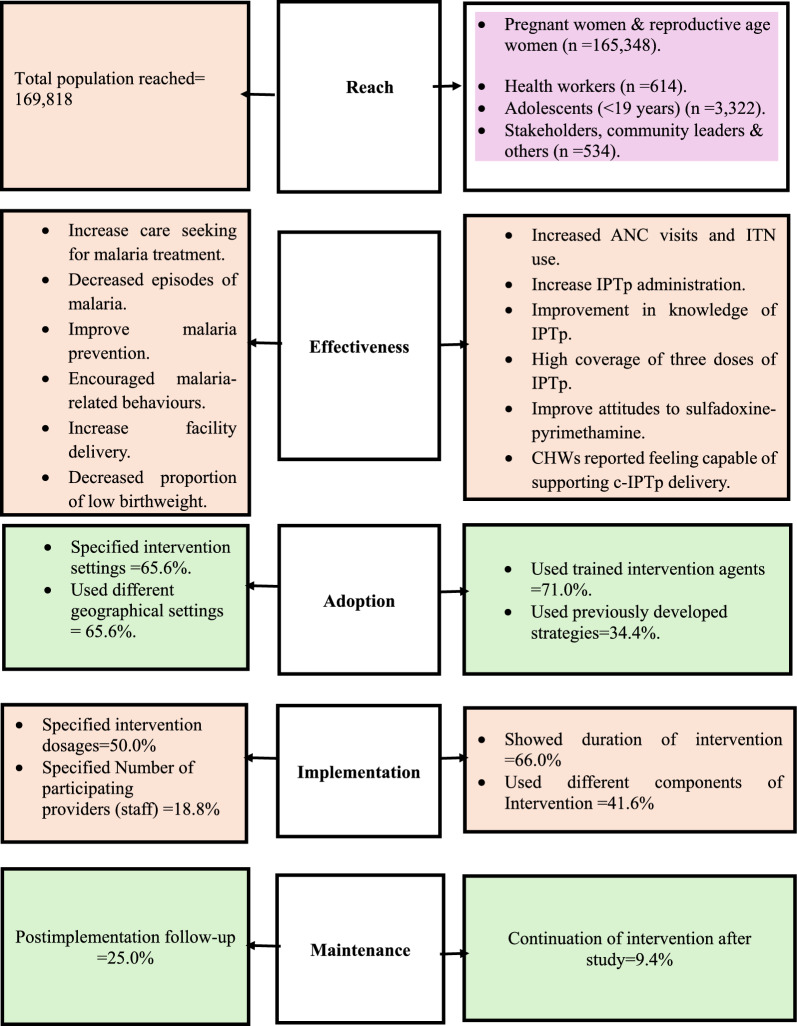
RE-AIM framework for IPTp intervention

IPTp coverage varied significantly among the studies and populations. Twenty-two (68.8%) of the studies reported coverage of ITPp among the populations studied, whereas 13 (59.1%) reported coverage of IPTp before and after the interventions. Nine (40.1%) of the twenty-two studies reported overall coverage of IPTp [16, 29–36], ranging from 9.3% to 94.4% for IPTp3, 21% to 92% for IPTp2 and 34% to 95% for IPTp1. Studies that were conducted in Guinea, 94.4% [37], Uganda, 85.8% [38], Malawi, 81% [39] and Kenya, 77.3% [34], reported a higher proportion of IPTp coverage. Most studies (37.5%) used at least three doses of IPTp as an outcome for the assessment, and 8 (25%) studies relied on at least two doses. The studies reported varying degrees of reporting on the various interventions used across the RE-AIM dimensions (Table 2 and Fig. 4).

**Table 2:** RE-AIM criteria included in each study

	Reach			Effectiveness			Adaptation				Implementation				Maintenance	
Study	Developed a defined Sample size	Described target population	Showed secondary outcome	Showed increase in result after Intervention	Used a control group	Developed an inclusion/exclusion criteria of the setting	specified intervention settings	Used different geographical settings	Used trained intervention agents	Used previously developed strategies	Specified intervention dosages	Specified Number of participating providers (staff)	Showed duration of intervention	Used different components of Intervention	Post implementation follow-up	Continuation of intervention after study
Hildon et al., 2020 [13]		*	*	*			*		*				*			*
Hoyt et al., 2018 [14]	*	*		*	*		*	*		*	*	*	*			
Msyamboza et al., 2009 [16]		*	*	*	*		*		*	*		*	*			*
Enguita-Fernàndez et al., 2021 [27]		*	*	*	*			*	*		*		*			*
Koita et al., 2024 [29]	*	*	*	*					*				*			
Grietens et al., 2010 [30]	*	*	*	*					*		*		*	*		
Vanga-Bosson et al., 2011 [31]	*	*	*	*		*			*	*			*	*		
Mbonye et al. 2008 [32]		*	*	*		*	*		*					*	*	*
Odwe et al., 2023 [33]		*		*		*				*			*			
Noguchi et al., 2020 [34]	*	*	*	*	*	*	*	*	*		*		*			
Koné et al., 2023 [35]	*		*	*	*		*	*	*	*			*	*		*
Gies et al., 2009 [36]			*	*		*					*		*	*		
Flueckiger et al., 2019 [37]		*	*	*	*	*			*		*		*			
Rassi et al. 2018 [38]	*	*	*	*			*		*		*			*	*	
Rubenstein et al., 2022 [39]	*	*	*			*			*				*			
Winskill et al., 2019 [40]			*	*			*			*						
Ouédraogo et al., 2022 [41]	*	*		*	*	*	*		*				*		*	*
Okeibunor et al., 2011 [42]			*	*			*		*							
Doumbia et al., 2021 [43]		*	*	*			*		*	*	*			*		*
Gutman et al. 2020 [44]		*	*	*	*	*	*		*				*			*
Mbonye, Bygbjerg, & Magnussen, 2007 [45]		*		*			*		*		*		*			*
Gies et al., 2008) [46]	*	*		*	*		*		*		*	*				
Ouma et al., 2007 [47]	*	*	*	*	*		*	*	*		*		*			
Wolf et al., 2023 [48]	*		*	*				*		*			*			
Mens, 2011 [49]		*	*	*			*		*	*		*		*		*
Burke et al., 2021 [50]				*										*		
Orobaton et al., 2016 [51]		*	*	*		*	*		*		*		*	*		
Graham & Ba-Break., 2013 [52]	*	*		*			*		*		*	*	*			*
Balami et al., 2019 [56]		*		*	*	*		*	*		*		*	*		
Faye & Lugand, 2021 [57]	*	*		*	*		*	*	*	*	*		*	*		*
Mbonye et al., 2013 [60]	*		*	*	*		*		*		*			*		
Hansen et al., 2012 [61]		*				*				*			*			
Total	15	26	21	30	14	13	21	8	26	12	16	6	25	14	3	13

* indicates the presence of a specified RE-AIM indicator for the article

### Reach

The reach dimension assesses the extent to which the intervention impacts the target population. The number and characteristics of individuals who participated in interventions for the included articles were assessed to determine the impact on the target population. The reach dimension was examined across three distinct areas: sample size, target population, and primary and secondary outcomes. These elements assessed refer to the parameters included in the study evaluating the project implemented, not the project itself. These elements are critical for understanding the scale and inclusiveness of the intervention’s evaluation. For example, the reach dimension in a study would analyse the number and characteristics of participants (e.g., pregnant women, health workers) who were involved in or impacted by the intervention during the project's implementation phase. The results showed that almost 96.9% of the articles reported the sample size, target population and outcomes, with the exception of one study (3.1%) [40]. The inclusion and exclusion criteria were reported in all studies, while the sample size was reported in all except one study, ranging between 15 and 169,818 participants. The ages of the participants ranged from 14 to 40 years. A total of 169,818 individuals belonging to various subpopulations, including pregnant women (165,348), adolescents (3,322), health workers and health managers including males and females (614), community leaders, stakeholders, and key influencers (534), were reached. Additionally, 72% of the 32 articles specified the target population and provided a thorough description. However, the socioeconomic status of the participants was not reported in all interventions, as the population studied was diverse across all studies. An assessment of the intervention's effectiveness demonstrated enhanced uptake of IPTp dosages and increased attendance at ANC visits.

### Efficacy/effectiveness

A greater proportion (87.5%) of the studies reported an increase in IPTp uptake following the interventions, whereas one study [41] reported slightly lower outcomes after the intervention. Before the interventions, IPTp coverage across the studies ranged between 0% and 55.1%, with postintervention coverage ranging from 2.1% to 85.8% [16, 34, 36, 38, 39, 42, 43]. Other secondary outcomes included increased care seeking malaria treatment, improved malaria-related behaviours, increased facility delivery, increased IPTp administration, increased ANC visits and ITN use, improved knowledge of IPTp, high coverage of three doses of IPTp, a decreased proportion of low birth weight, and decreased episodes of malaria.

### Adoption

The evaluation of the adoption dimension aimed to determine whether there was a specific setting, and the characteristics of the setting intervention were outlined. The criteria of interest for this dimension included whether the intervention was conducted in various or specific geographical locations. A total of 65.6% of the articles involved a specific setting, including localities with low IPTp coverage [38], areas with active CHWs [44], and malaria hyperendemic locations [33, 41, 45–47], whereas 71% of the interventions employed trained intervention agents. Additionally, 34.4% of the interventions used a previously designed strategy, including training manuals on malaria [38] and interventions targeting populations in low-resource settings [35].

### Implementation

The practical application and integration of evidence-based interventions are emphasized in the implementation component of the RE-AIM framework. The specified articles illustrated this by measuring the number of times the intervention was administered to the group of interest, with 50% of the articles indicating the specific number of times it was administered. Furthermore, 18.8% of the intervention articles mentioned the number of agents, including gynecologists [43], trial staff [14], midwives and other community health workers locations [38, 44, 60] and female community leaders (women field assistants (WFAs) [46] who carried out the interventions, whereas 66% mentioned the duration of the intervention locations [37, 45, 48]. Approximately eight hundred and twenty-three trained agents were used. Finally, 40% of the articles utilized different components of the intervention strategy as if they had distinct components. Importantly, these findings were based on a specific set of articles and may not be representative of all interventions in the field. Intervention strategies that were implemented and yielded positive results include health education on malaria [16, 47], door-to-door campaigns on IPTp [49] and complementary community-directed intervention (CDI) programs [42].

### Maintenance

The maintenance component of the RE-AIM framework focuses on the sustainability and long-term impact of the interventions. Unfortunately, this aspect has been the least reported in the literature, with only 9% of the studies providing postimplementation follow-up. Approximately 40.6% of the interventions, however, continued after the study period.

### Gender analysis of IPTp interventions

Table 3 highlights the gender dynamics of different interventions via the gender analysis framework adapted from Morgan et al*.* [28]. Some studies [13, 30, 32, 50] have inherently considered gender, particularly in interventions related to women’s reproductive health. For instance, Grietens et al*.* [30] demonstrated that adolescent girls’ social roles and labour demands negatively affected their access to IPTp and other healthcare services during pregnancy. Therefore, when interventions are designed from a gender perspective, they help dismantle structural barriers and social norms, improving adolescents’ access to pregnancy-related healthcare (see also [13]). In addition, involving both men and women in health interventions was found to promote gender-equal roles, attitudes, and uptake of malaria prevention practices [13].

**Table 3:** Gender dynamics of the various interventions

Article	Access To Resources Needed for IPTp Treatment	Roles And Practices Affecting IPTp Uptake	Social Norms, Values and Beliefs Affecting IPTp Uptake	Decision-Making Power, Autonomy of IPTp Uptake
Effect of a community-based delivery of intermittent preventive treatment of malaria in pregnancy on treatment seeking for malaria at health units in Uganda [32].	Women's treatment choices often influenced by men due to financial control			Men's decisions crucial in seeking care for sick children and women
Community-based delivery of intermittent preventive treatment of malaria in pregnancy in Burkina Faso: A qualitative study [50].			Cultural limitations to male community health workers interactions with pregnant women whose husbands may not be comfortable with a male health worker making home visits	
Intermittent preventive treatment of malaria in pregnancy: Evaluation of a new delivery approach and policy implications for malaria control in Uganda [45].	Women reported getting resources from husbands' encouragement and supported them	Husbands supported women in accessing malaria prevention interventions. Some women had barriers like household work and fear of judgement		Family dynamics plays a crucial role in decisions on treatment and prevention
Women’s empowerment and uptake of sulfadoxine–pyrimethamine for intermittent preventive treatment of malaria during pregnancy: results from a cross-sectional baseline survey in the Lake endemic region, Kenya [33].	Women with high decision-making autonomy have higher odds of receiving 3 + doses of IPTp-SP during pregnancy	Men's involvement in health promotion and malaria prevention was encouraged	Women's decision-making power, control of assets, and educational attainment influence the uptake of IPTp-SP during pregnancy	1.Women with high decision-making autonomy had higher odds of taking 3 + doses of IPTp-SP 2. Decision-making autonomy enhances women's agency, giving them control over maternal health service decisions, including the uptake of interventions like IPTp-SP
Bottlenecks for High Coverage of Intermittent Preventive Treatment in Pregnancy: The Case of Adolescent Pregnancies in Rural Burkina Faso [30].	Married female adolescents have restricted mobility affecting healthcare access	Adolescents face structural constraints and household labor requirements	Structural social constraints like social position and labor requirements impact access of adolescents	Newly married adolescents have limited bargaining power due to gender roles
We have this, with my husband, we live in harmony”: exploring the gendered decision-making matrix for malaria prevention and treatment in Nampula Province, Mozambique [13].		Gendered decision-making matrix influenced by TTSM program for malaria prevention	Social norms, family hierarchy, and gender dynamics impact decision-making power	
Using Short Message Service Alerts to Increase Antenatal Care and Malaria Prevention: Findings from Implementation Research Pilot in Guinea [37].	Women with mobile phones were more likely to attend ANC visits			
Scaling-up the use of sulfadoxine pyrimethamine for the preventive treatment of malaria in pregnancy: results and lessons on scalability, costs, and programme impact [51].		Inequalities in power between genders can hinder women's health access	Social diffusion influenced IPTp-SP scale-up through public testimonies	Social factors impact women's decision-making power in accessing healthcare services
Trust, community health workers, and delivery of intermittent preventive treatment of malaria in pregnancy: A comparative qualitative analysis of four sub-Saharan countries [27].		Gender roles influence trust in female community health workers 2.Gender roles not considered in policies, impacting trust in CHWs	Policies often prioritize male CHWs, impacting community trust CHWs' competence is linked to gender expectations in healthcare	

It was observed that some studies [13, 51] indirectly addressed gender-related concerns, with 9 (28.1%) of the articles identifying the unequal or equal opportunities and challenges faced by pregnant women and adolescent girls in accessing IPTp interventions. Additionally, six studies [30, 32, 33, 37, 45, 50] examined women's access to the resources necessary for IPTp treatment. The results suggest that resource access is the major determinant of healthcare access, and when such resources are lacking, pregnant women and adolescent girls are unable to fund the cost of transportation and treatment [51].

On the side of health workers, resources are needed to ensure adequate supervision and distribution of IPTp services, but this is often constrained by the difficulty in accessing transportation resources and equipment [50]. To ensure that health workers seize every opportunity to provide SP to pregnant women, there is a need to equip them with adequate resources, essential equipment, and appropriate skills training [52, 53].

In addition, it was found that gender roles and practices impact IPTp uptake, which was documented by six of the articles [13, 27, 30, 32, 33, 45, 51]. In rural Burkina Faso, for example, [30] showed that owing to gender roles and stricter social expectations such as working hard, respecting in-laws, bearing children, and taking on additional domestic tasks related to both intra- and interhousehold labour distribution, newly married adolescent girls have limited bargaining power, which poses an enormous challenge in reaching this age group to provide SP during the malaria transmission season.

In some studies [13, 27, 30, 33, 50, 51], social norms, values, and beliefs about gender influence the uptake of IPTp, whereas issues of decision-making power and autonomy regarding IPTp uptake also affect malaria prevention efforts [30, 32, 33, 45, 51]. Most of the studies (20 out of 32) focused on pregnant women, adolescents or children under five years of age, who are at increased risk of malaria infection.

## Discussion

The impacts of the intervention programmes (i.e., reach and efficacy/effectiveness) were well reported across the studies. Overall, a total of 169,818 populations comprising different subpopulation groups, including pregnant women, adolescents, children under five, health workers, community members and key informants, were reached. Most of the interventions were designed to reach pregnant women and adolescents ranging in age from 15 to 50 years. An evaluation of the intervention’s effectiveness revealed improved outcomes for IPTp dosage uptake and ANC visits.

Using the RE-AIM framework, interventions that aimed to enhance the adoption of IPTp treatment were reviewed. Most studies reported well on the impact of the intervention programs through the reach and effectiveness criteria. However, there was limited information provided about the institutionalization of the programs, such as adoption and implementation, and the maintenance of the intervention programmes. This lack of information limits the generalizability of the outcomes of intervention programmes, which is consistent with other reviews that have used the RE-AIM framework [54, 55]. Most of the intervention programs aimed to educate communities and promote assistance for pregnant women in obtaining IPTp treatment, resulting in an increase in uptake after interventions [56]. The effectiveness of interventions to further increase IPTp uptake varied among the reviewed articles. Some articles reported the use of training and guidelines for health workers as well as CHWs as key factors leading to an increase in IPTp uptake. Others emphasized the importance of providing training to healthcare workers [27, 57], as well as the implementation of a revised national guideline for IPTp treatment [27]. Building trust between health workers and the community helped increase the uptake of IPTp by providing training on integrating communities with the formal healthcare system through strategies involving CHWs [27]. This contributed to building trust in the CHWs’ performance and the involvement of the community.

### Gender advocacy

Access to resources needed for treatment, transportation to clinics and financing medical costs, among other needs, influences women's uptake of IPTp to prevent malaria. Previous studies [13, 30, 32–34, 45] highlighted the need for women to have access to resources and how that could help improve IPTp uptake. Men often influence women’s treatment choices owing to financial restraints caused by control over women’s financial resources [58]. Women with access to resources showed significant decision-making autonomy and thus had higher odds of receiving 3 + doses of IPTp-SP during pregnancy [33]. Furthermore, women with mobile phones were more likely to attend ANC visits. They received IPTp treatment, as reported in a pilot study conducted using short-message service alerts to increase antenatal care and malaria prevention during an intervention [37]. Notably, some of the women reported that the resources from their husbands, encouragement, and support during the study had increased, thus showing an increase in the involvement of men in IPTp interventions, thus correlating with increased SP usage [45].

Gender roles and practices significantly affect IPTp uptake [33]. Inequalities in power dynamics and gender roles were, for instance, found to hinder women’s access to health [33]. In addition, some women reported concerns, such as domestic responsibilities and fear of judgment for not performing their expected gender roles as barriers to accessing ANC services to take SP. A study by [30] also reported that adolescents in the intervention faced structural constraints and household labour requirements, including being hardworking, respecting in-laws, bearing children, and taking on additional domestic tasks related to both intra- and interhousehold labour distribution. These constraints make adolescents highly vulnerable during their pregnancies, particularly during the high malaria transmission season.

Women's socioeconomic and demographic characteristics, including their age, education, and marital status, were also found to influence their adherence to IPTp and delivery to health facilities [59]. Gender also influenced trust among female CHWs. In one intervention study, for example, policies that did not consider gender or policies that were gender-blind affected trust in CHWs delivering IPTp and essentially acceptability and adherence to the intervention [60]. Addressing gender dynamics, empowering women, building trust in healthcare providers, and enhancing gender-sensitive community-based interventions are vital strategies for improving IPTp uptake and maternal health outcomes. For example, gender norms influence perceptions of competence in CHWs, with female CHWs often being more capable of providing health services related to pregnancy, indicating a greater sense of trust between women and female CHWs [27] while advocating for better perceptions of male CHWs.

Social norms and values also influence IPTp uptake. Low socioeconomic status, high parity, and unplanned pregnancies are barriers affecting access to IPTp [41]. Cultural norms and social factors such as shame and embarrassment around adolescent pregnancies hinder early ANC attendance, impacting access to interventions, such as IPTp-SP [30]. A study on adolescent pregnant women’s uptake of IPTp reported that patrilocal residence patterns limited adolescents' autonomy and bargaining power, affecting their ability to engage in health-promoting activities, such as attending antenatal care (see also [30]. Therefore, engaging husbands or male household heads in decision-making and promoting supportive roles could enhance women's agency in malaria prevention, contributing to the improved uptake of interventions.

Women's decision-making autonomy is often measured by their involvement in decisions on personal earnings, healthcare, household purchases, and family visits [33]. The study by [33] highlighted the positive impact of women’s decision-making power and autonomy on IPTp-SP adoption during pregnancy. The utilization of a gender decision-making matrix has been demonstrated to affect the selection of preventive and therapeutic alternatives [13]. Numerous reports have indicated a consistent connection between a woman’s ability to make decisions and her use of maternal health services, highlighting the significance of empowerment in health care choices. It is essential to consider these factors when analysing decision-making processes to ensure a comprehensive understanding of women’s autonomy and empowerment within various societal contexts [51]. Higher levels of education are correlated with increased acceptance of IPTp-SP, highlighting the connection between education and the utilization of maternal health services that aim to empower women, particularly those with limited decision-making power and educational attainment [33, 58]. Thus, women’s decision-making power is crucial to IPTp uptake.

Generally, the findings in this review highlight several important implications for improving IPTp uptake and reducing malaria-related risks during pregnancy. It was found that addressing gender dynamics and enhancing community engagement are critical strategies to overcome existing barriers and foster sustainable progress. The paper observed that many women and adolescent girls face significant barriers in accessing health interventions like IPTp. These barriers range from social and cultural norms and limited decision-making autonomy to resource constraints including financial and transportation resources. It is envisaged that interventions that address these structural barriers and targeted support for vulnerable groups including adolescent girls and women in low-resource settings, can significantly increase IPTp uptake.

Additionally, the review highlights that community-based interventions play a key role in improving the reach and adoption of IPTp. Community engagement enhances awareness, trust, and participation, which are critical for reducing barriers [27]. Therefore, addressing gender dynamics and strengthening community engagement can contribute to better ANC attendance, higher IPTp adherence, and reduced malaria prevalence during pregnancy. A reduction in malaria-related risks in pregnancy can result in improvement in neonatal health outcomes, including reduced low birth weight and infant mortality rates.

## Conclusion

In conclusion, addressing gender dynamics and enhancing community engagement is crucial for increasing IPTp uptake and reducing malaria-related risks during pregnancy. Sustainable, integrated and gender-inclusive strategies are essential for progress towards malaria elimination by 2050. CHWs can be mobilized to deliver malaria prevention and control measures, while women are empowered to participate in health decision-making to improve IPTp uptake. It is recommended that women are empowered through education and resources to make informed health decisions, whereas men are encouraged in health interventions to support women's access to treatment. The paper envisaged that the utilization of gender-sensitive approaches in community-based interventions can improve IPTp adherence.

### Strengths of the study

This review provides a complete evaluation of the effects of integrated strategies, including community health worker involvement in IPTp promotion, community-based delivery, health worker training, and other interventions, on the uptake of IPTp-SP in preventing malaria infection during pregnancy. Using the RE-AIM framework helped in understanding the effectiveness of each adopted intervention and the population subgroups that these interventions reached. Additionally, the strength and added value of this study lies in its application of the gender analysis framework, which highlights the significance of gender norms and sociocultural barriers associated with the administration and uptake of IPTp-SP for malaria prevention during pregnancy.

## Data Availability

No datasets were generated or analysed during the current study.

## Abbreviations

CCM: Community Case Management LMICs
CHWs: Community health workers
IPTp: Intermittent preventive treatment of malaria in pregnancy
IPTp-SP: Intermittent preventive treatment of malaria during pregnancy with sulfadoxine-pyrimethamine
ITN: Insecticide-treated nets
MiP: Malaria in pregnancy
PRISMA-ScR: Preferred reporting items for systematic reviews and meta-analysis extension for scoping reviews
RE-AIM: Reach, effectiveness, adoption, implementation, and maintenance
SSA: Sub-Saharan African
WHO: World Health Organization
